# Blunt Isolated Small Bowel Perforation Intervention: Does a Delay in Management Matter?

**DOI:** 10.1155/2020/7478485

**Published:** 2020-06-04

**Authors:** Sung Yong Hong, Se Hun Kim, Ki Hoon Kim

**Affiliations:** ^1^Department of Critical Care Medicine, Haeundae Paik Hospital, Inje University College of Medicine, Busan, Republic of Korea; ^2^Department of Anesthesiology, Haeundae Paik Hospital, Inje University College of Medicine, Busan, Republic of Korea; ^3^Department of Surgery, Haeundae Paik Hospital, Inje University College of Medicine, Busan, Republic of Korea

## Abstract

**Purpose:**

Blunt small bowel injury is rare, and its timely diagnosis may be difficult. The effects of a delayed intervention on prognosis are unclear. We aimed to determine whether the time to surgical intervention affects outcomes in patients with blunt small bowel perforation.

**Methods:**

The study was performed between March 2010 and December 2018 in adults (age >18 years) who initially underwent computed tomography and small bowel surgery only and survived more than one day postoperatively. They were categorized into three groups based on injury-to-surgery time intervals: ≤8, 8–24, and >24 h; similarly, they were also categorized into two groups of ≤24 and >24 h.

**Results:**

Bowel resection, length of stay (LOS), intensive care unit (ICU) LOS, morbidity, and mortality were analyzed as outcomes in 52 patients. The number of patients in the three groups (≤8, 8–24, and >24 h) based on the time-to-surgery was 33, 13, and 6, respectively. On comparing the three groups, there were no significant differences in LOS (24 [18–35], 21 [10–40], and 28 [20–98] days, respectively; *p*=0.321), ICU LOS (2 [1–12], 4 [2–26], and 11 [7–14] days; respectively, *p*=0.153), mortality (3% (*n* = 1), 15% (*n* = 2), and 0%, respectively; *p*=0.291), and morbidity (46% (*n* = 15), 39% (*n* = 5), and 50% (*n* = 3), respectively; *p*=0.871). However, there was a significant difference between the groups in bowel resection (67% (*n* = 22), 31% (*n* = 4), and 83% (*n* = 5), respectively; *p*=0.037). Additionally, there was no significant difference in outcomes between the two groups (≤24 and >24 h) with small bowel perforation.

**Conclusions:**

Delay in surgical intervention following blunt abdominal trauma may not affect the outcomes of patients with small bowel injuries, such as LOS, ICU LOS, morbidity, and mortality, except bowel resection.

## 1. Introduction

Small bowel injury is the third most common injury in patients with blunt abdominal trauma. A previous multicentric study reported that it was diagnosed in 1.1% of patients admitted with a history of blunt abdominal trauma; of these, only 0.3% were diagnosed with small bowel perforation [1]. Another recent study has reported that small bowel perforation was present in only 0.06% and 0.08% of admissions and emergency activation for trauma care, respectively [2].

Examinations, including abdominal X-rays, ultrasonography, peritoneal lavage, computed tomography (CT), and exploratory laparoscopy, have been traditionally used to evaluate the extent of injuries following blunt abdominal trauma [3–5]. CT is the currently accepted standard imaging modality, not only for the diagnosis of abdominal solid organ injury but also for evaluating suspected trauma involving the hollow viscera in an otherwise hemodynamically stable patient [3–6]. The abdominal radiographic findings that most often correlate with diagnosis of small bowel injury include the presence of free intraperitoneal gas or fluid, enhanced bowel-wall thickening, mesenteric fat stranding, and detection of extraluminal gas [7, 8]. However, some patients demonstrate fewer or no abnormalities on the initial CT scans. In a systematic review of multidetector CT scans performed for evaluating bowel injuries with or without mesenteric involvement following blunt trauma, the sensitivity and specificity were 87–95% and 48–84%, respectively [9, 10]. Therefore, an initial CT scan failed to detect approximately 15% of small bowel injures [11, 12]. The reliability of CT in identifying blunt injury to the bowel, which requires surgical repair, is controversial.

The low incidence of small bowel injury and difficulty in diagnosis can result in delayed surgical interventions. A diagnostic delay of more than 24 h correspondingly increases morbidity and mortality due to injury [1]. Several studies have suggested an association between a diagnostic waiting time of <5–8 h and higher morbidity and mortality [13, 14]. However, other reports revealed that a delay in either diagnosis or treatment of blunt bowel injury does not correlate with an increased incidence of subsequent complications [15–17]. This study aimed to determine whether the time between injury and surgery had any effect on the outcomes of patients who underwent surgery for blunt small bowel perforation. We not only performed a comparative analysis based on an 8 h delay but also evaluated the outcomes in patients who underwent surgery after a 24 h interval. We hypothesized that a delay in treatment could be associated with an increased risk of subsequent morbidity or mortality.

## 2. Materials and Methods

**Figure 1:**
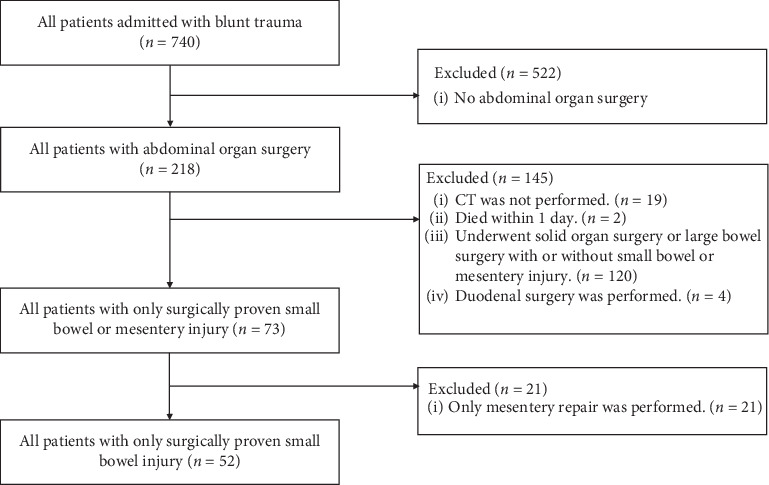
Flow diagram showing patients with blunt abdominal trauma.

The data of all adult patients who presented with blunt abdominal injury were identified in the trauma database of Haeundae Paik Hospital between March 2010 and December 2018 and enrolled in the study. The inclusion criteria were age >18 years and admission to the emergency department for blunt abdominal trauma during the study period. Patients who did not undergo abdominal organ surgery, died within a day of admission, underwent other abdominal organ surgeries (solid organ, duodenum, large bowel) in the presence or absence of associated small bowel injury or mesenteric injury, and underwent only mesenteric repair and in whom abdominopelvic CT scan was not performed on admission to the emergency department were excluded from the study (Figure 1). Finally, the enrolled patients were categorized into three groups based on injury-to-surgery time intervals: ≤8, 8–24, and >24 h. Additionally, the outcomes were also analyzed by categorization into two groups of ≤24 and >24 h of injury-to-surgery time interval. Relevant factors were compared between the groups. We analyzed the presence of bowel resection, length of stay (LOS), intensive care unit (ICU) LOS, mortality, and morbidity, as patient outcomes.

All the patients' hospital charts, as well as their data from the database, were reviewed and analyzed. The following patient's data were identified, collated, and analyzed: demographic characteristics, mechanism of injury, admission Glasgow coma scale (GCS) score, injury-to-operating room time, emergency department-to-operating room time, abbreviated injury scale (AIS) code, injury severity score (ISS), clinical examination findings, laboratory and CT results, bowel injury prediction score (BIPS), whether small bowel resection was performed during operative abdominal exploration, LOS, ICU LOS, mortality, and morbidity. Mortality was defined as death occurring at any time during the entire LOS after the first 24 h of hospitalization. The recorded clinical findings included heart rate, systolic blood pressure, and abdominal pain. Pain was defined as spontaneous pain, tenderness, or rigidity, elicited only on abdominal palpation, and was considered absent if the patient was uncooperative or unconscious during the physical examination. Laboratory examination results included hemoglobin levels, particularly the lowest level recorded during the first 24 hours, white blood cells (WBC) count, and C-reactive protein (CRP) levels. Significant CT findings included the detection of bowel-wall thickening, mesenteric stranding, intra-abdominal free air or fluid, and intravenous contrast extravasation. All available clinical data, laboratory results, and CT images were collated from the data recorded during the initial phase of care in the emergency department. A contrast-enhanced CT scan (CECT) was preferentially performed in hemodynamically stable patients and in those assessed by the staff as being able to tolerate the examination, even when they were hemodynamically unstable. BIPS is a novel predictive score for bowel injuries and was first introduced by McNutt et al. [18]. Using this score, the clinician can predict the risk of bowel injury when two or more of the following parameters are detected in the emergency department: (i) high CT grading scale (≥4) for mesenteric injury, (ii) elevated WBC count (≥17,000/*μ*L), and (iii) the presence of abdominal pain. High CT grade for mesenteric injury was considered as the presence of a mesenteric contusion or hematoma with associated bowel-wall thickening and adjacent interloop fluid collection, or active vascular/oral contrast extravasation, bowel transection, or pneumoperitoneum [18]. This study was approved by the Institutional Review Board of Inje University Haeundae Paik Hospital (HPIRB 2019-05-002-001). This trial is registered with KCT0004644.

### 2.1. Statistical Analysis

Nonnormally distributed variables are expressed as median and interquartile range (IQR) and normally distributed variables as mean and standard deviation (SD) for continuous variables. Categorical data are described as count and percentage. The Shapiro–Wilk test was performed to verify the assumption of normality. After descriptive analyses were performed, chi-square or Fisher's exact test was used to compare categorical variables between the groups, and the independent *t*-test or Mann–Whitney *U* test was used to compare continuous variables between the groups. Intergroup differences in nonparametric variables were compared using the Kruskal–Wallis test with Tukey corrected post hoc tests, and they were analyzed using a one-way analysis of variance with Bonferroni corrected post hoc tests for parametric variables. A difference was considered significant if the 2-tailed *p* value was <0.05. Data analysis was performed using SPSS v25 (IBM Inc., Armonk, NY, USA).

## 3. Results

**Figure 2:**
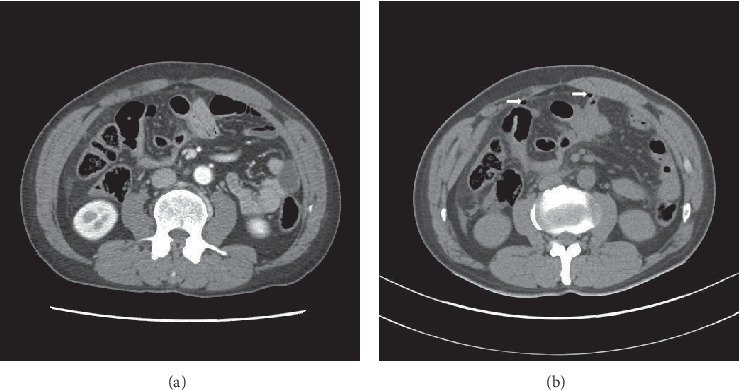
A 40-year-old man with blunt abdominal trauma due to assault. (a) Axial contrast-enhanced CT scan shows focal small bowel ileus in the LUQ. (b) Follow-up CT scan was performed after 4 hours. Axial unenhanced CT scan shows small air bubbles (white arrow) between the bowel lumen and the peritoneal cavity.

A total of 740 patients with a history of blunt abdominal trauma were admitted during the study period. Out of these, 218 patients underwent abdominal surgeries. 166 patients were excluded from the study group, and the remaining 52 patients' data were finally analyzed (Figure 1). Out of these 52, nine patients were found to have bowel injury on repeated CT scans that was confirmed intraoperatively (Figure 2). The median time interval before performing a repeat CT scan was 22 h (range, 6.5–38.5). The demographic characteristics of all patients are summarized in Table 1. In this cohort, 73% were males. The overall median time intervals from injury to surgical intervention and from admission to the emergency department to transfer to the operating room were 5.8 h (range, 3.8–10.7) and 2.9 h (range, 2.0–6.5), respectively. The causes of injury were classified into two main groups: motor vehicle collisions (*n* = 23, 44%) and others (*n* = 12, 23%). The initial CT findings demonstrated intra-abdominal free air and fluid in 73% and 90% of all patients, respectively. The number of patients in the three groups (≤8, 8–24, and >24 h) based on the time-to-surgery was 33, 13, and 6, respectively. While comparing baseline variables between the three groups (Table 1), we found significant differences in the injury-to-operating room time (*p* < 0.001), emergency department-to-operating room time (*p* < 0.001), and initial hemoglobin level (*p*=0.040).  Table 2 summarizes the comparisons of the treatment outcomes according to the time-to-surgery. Small bowel resection was performed in 60% of all patients (*n* = 31), and primary repair of small bowel was performed in 40% of patients. The overall median LOS and ICU LOS were 24 days (range, 17–36) and 4 days (range, 1–12), respectively. Overall, three patients died after surgery (mortality rate, 6%). The overall morbidity rate was 44% (23 patients), with a wound complication rate estimated at 31%, making it the commonest postoperative complication. In the comparative analysis of the three groups based on the injury-to-surgery time, a statistical difference in bowel resection was observed (*p*=0.037). However, there were no significant differences in LOS, ICU LOS, mortality, and morbidity among the groups. Furthermore, a difference in bowel resection was not observed in the post hoc analysis.

Table 3 summarizes the comparisons of outcomes between the two groups (≤24 and >24 h) with small bowel perforation. The presence of free air on CT was more in the group treated within 24 h than the group treated after 24 h (*p*=0.038). Although mortality was observed only in patients who underwent surgery within 24 h (7%), the mortality rate did not differ significantly (*p*=1.000) between the two groups.

## 4. Discussion

We retrospectively analyzed data of 52 patients who underwent surgery for small bowel injury following blunt abdominal trauma. We found no statistically significant differences in ICU LOS, mortality, and morbidity among patients with different injury-to-surgery times, but only in three groups of comparative analysis based on an 8 h interval, we found a statistical difference in bowel resection.

Diagnostic confirmation of hollow visceral injuries is difficult compared to those involving solid organs, especially in patients with polytrauma who present with associated head and spinal cord injuries or with altered consciousness [12, 16]. The delayed diagnosis of bowel perforation may lead to peritonitis due to spillage of bowel contents, and it may adversely affect the prognosis of patients with small bowel perforation. Several studies have reported that delayed diagnosis or treatment of this type of injury could be associated with increased mortality or morbidity. Faria et al. [19], in their study of 102 patients with bowel injuries, noted that all postoperative deaths occurred in patients who underwent surgery after the first 24 hours. Another large study sponsored by the EAST Multi-Institutional HVI Research Group found that a delay in diagnosis of small bowel perforation of >24 hours led to increased morbidity and mortality. Mortality from a hollow viscus injury was demonstrated to increase from 13% to 30.8% in patients who underwent surgery at <8 h and after 24 h, respectively [1]. Additionally, the delay in surgery was found to result in higher morbidity, including sequelae such as wound infection, wound dehiscence, and intra-abdominal abscess formation [1]. Fakhry et al., in a multicenter study of 198 patients with small bowel injury following blunt abdominal trauma, reported that mortality rates increased with increasing time interval to surgical intervention (2%, 9.1%, 16.7%, and 30.8% for time-to-surgery groups of <8, 8–16, 16–24, and >24 h, respectively; *p*=0.009) [13]. Therefore, the results of their study indicated that even relatively brief delays (≤8 hours) could result in morbidity and mortality. Al-Hassani et al. have also reported an increase in the incidence of morbidity with operative delay >8 hours after a comparison between two groups of surgically treated patients—those who underwent surgery within and beyond 8 h of sustaining injury [20]. Another study by Malinoski et al. demonstrated increased mortality rates in patients with an operative delay of only >5 h [14].

Conversely, some studies have reported no association between operative delay for blunt small bowel injury and consequent morbidity and mortality. Fraga et al., in their comparative study of patients with isolated small bowel injury with those also having other associated injuries, demonstrated that the diagnosis was commonly delayed in the former group [16]. However, the rates of complications did not differ significantly between patients with perforated small bowel injury who underwent repair within 6 h interval and those who underwent surgeries after 6 h. In a systematic review of 11 studies on the clinical outcomes of delayed intervention in patients with hollow visceral injuries due to blunt abdominal trauma, Harmston et al. reported that while 10 studies demonstrated no difference in mortality, four others reported a significant difference [17]. Ahmed and Greenberg conducted a study examining the effects of time interval from sustaining an injury to receiving a small bowel resection procedure on the outcomes of patients with blunt abdominal trauma using a propensity-matched analysis [21]. Their study demonstrated no statistically significant difference in the mortality rates in a comparative analysis between two groups of patients operated at <4 and >4 h (8.3% vs. 7.9%, respectively; *p*=0.90). The present study results also demonstrate that morbidity and mortality are not associated with a delay in the surgical intervention [21].

As CT became the diagnostic imaging modality of choice, nonoperative management has taken precedence in the management of patients with abdominal solid organ injuries. This has led to a significantly larger proportion of patients never undergoing a laparotomy following abdominal trauma [17, 22]. Given these changing trends in the management of blunt abdominal trauma, both physical examination and CT scan findings have become more important as a collective tool for the diagnosis of hollow viscera injuries. Physical examination, however, may not be diagnostically helpful if an associated brain or spinal cord injury is present. Additionally, although the accuracy of CT in the diagnosis of blunt bowel injury has increased, it also results in false-negatives in 10–13% of patients with a perforated small bowel. Walker et al. reported that the early repetition of abdominal CT is useful in the detection of bowel perforation in patients with blunt abdominal trauma [23]. Saku et al. recommended performing a follow-up CT scan 8 h after an initial negative one to increase the investigation sensitivity in detecting small bowel injury, as the incidence of intra-abdominal extraluminal air-collection increases over time [24]. In our study, nine patients (17%) were diagnosed with a bowel injury after a repeat CT scan, and the median time interval between the initial and repeat investigation was 22 hours. This was longer than that in other studies because patients with multiple injuries were initially admitted to the neurosurgery or orthopedic surgical departments, thus, leading to a delayed diagnosis of their small bowel injuries.

This study has several limitations. First, the data were retrospectively collated from medical records and no randomization technique was applied. Second, the study included a small number of admitted patients in a single institution. Therefore, the small sample size may have reduced the statistical significance. Furthermore, the choice of surgical treatment of blunt bowel injury was dependent on the experience and preference of each treating surgeon, which resulted in variations in the management. Most surgeons had clinical experience of ≥5 years after acquiring their professional qualification; therefore, differences in clinical decision-making may have been minimal. Again, the diagnosis of small bowel injury was delayed due to late CT scan follow-up when patients with multiple injuries were initially admitted to the neurosurgery or orthopedic surgery departments. This may have affected the delayed intervention. To clarify these issues, future prospective, large-scale, and multicenter studies are required. In addition, as a follow-up, studies on the usefulness of repeat CT and the appropriate time intervals of follow-up CT are needed to reduce the likelihood of delayed bowel injury.

## 5. Conclusion

Delay in surgical intervention following blunt abdominal trauma may not affect the outcomes of patients with small bowel injury with regard to LOS, ICU LOS, morbidity, and mortality except for bowel resection. However, the number of patients who underwent surgery after more than 24 hours was quite small in this study. Therefore, multicenter studies with larger sample sizes are needed to validate our results.

## Figures and Tables

**Table 1:** Summary of baseline characteristics for the study population.

	All patients, *n* (%) (*n* = 52)	Time to operative intervention group			*p* value
		≤8 h (*n* = 33)^1^	>8 h to ≤ 24 h (*n* = 13)^2^	>24 h (*n* = 6)^3^	
Male	38 (73)	24 (73)	10 (77)	4 (67)	0.894
Age (y), mean ± SD	53 ± 16	53 ± 15	52 ± 19	61 ± 9	0.421
Abdominal pain	48 (92)	31 (94)	12 (92)	5 (83)	0.669
Time from injury to OR, median (IQR, 25–75)	5.8 (3.8–10.7)	3.5 (3–5)	10.8 (9.4–15.4)	43.8 (37.5–81)	<0.001^†^
Time from ER to OR, median (IQR, 25–75)	2.9 (2.0–6.5)	2.5 (2.0–3.7)	6.4 (3.3–12.9)	31.1 (4.3–43)	<0.001^†^
Injury mechanism					0.993
MVC	23 (44)	15 (46)	6 (45)	2 (33)	
MBC	6 (12)	4 (12)	1 (8)	1 (17)	
Pedestrian	6 (12)	4 (12)	1 (8)	1 (17)	
Falls	2 (4)	1 (3)	1 (8)	0 (0)	
Bicycle	3 (6)	2 (6)	1 (8)	0 (0)	
Others	12 (23)	7 (21)	3 (23)	2 (33)	
SBP (mmHg), median (IQR, 25–75)	109 (94–130)	105 (89–130)	120 (104–139)	103 (98–124)	0.524
Hypotension (SBP <90 mmHg)	10 (19)	8 (24)	2 (15)	0 (0)	0.352
HR (beats per minute), median (IQR, 25–75)	84 (75–98)	88 (70–98)	84 (80–93)	87 (78–104)	0.795
GCS, median (IQR, 25–75)	15 (14–15)	15 (15–15)	15 (15–15)	14 (14–15)	0.123
CT findings (initial)					
Bowel-wall thickness	45 (87)	30 (91)	11 (85)	4 (67)	0.270
Mesenteric stranding	46 (89)	28 (85)	12 (92)	6 (100)	0.498
Free air	38 (73)	26 (79)	10 (77)	2 (33)	0.065
Free fluid	47 (90)	30 (91)	11 (85)	6 (100)	0.564
Contrast extravasation	12 (23)	10 (30)	2 (15)	0 (0)	0.201
Initial Hgb (mg/dl), mean ± SD	13.4 ± 2.3	13.1 ± 2.4	14.7 ± 1.7	12.1 ± 1.8	0.040
Initial WBC (10^9^/L), mean ± SD	11.8 ± 5.4	12.0 ± 5.3	11.2 ± 4.7	12.1 ± 7.9	0.898
CRP (*n* = 44 mg/dL), median (IQR, 25–75)	0.24 (0.08–1.15)	0.16 (0.08–0.41) (*n* = 29)	0.89 (0.31–1.56) (*n* = 11)	10.77 (0.53–24.00) (*n* = 4)	0.138
Lowest Hgb (mg/dl), mean ± SD	10.3 ± 2.1	10.2 ± 2.2	10.6 ± 2.0	10.3 ± 1.6	0.865
Abdominal AIS score					0.143
3	35 (67)	19 (58)	11 (85)	5 (83)	
4	17 (33)	14 (42)	2 (15)	1 (17)	
ISS, median (IQR, 25–75)	18 (10–25)	18 (12–26)	14 (9–23)	19 (13–43)	0.289
BIPS					0.856
1	5 (10)	2 (6)	2 (15)	1 (17)	
2	38 (73)	25 (76)	9 (69)	4 (66)	
3	9 (17)	6 (18)	2 (15)	1 (17)	

SD standard deviation; OR operation room; ER emergency room; MVA motor vehicle collision; MBA motor bike collision; SBP systolic blood pressure in emergency department; HR heart rate in emergency department; GCS Glasgow coma scale; CT computed tomography; Hgb hemoglobin; WBC white blood cell; CRP C-reactive protein; AIS abbreviated injury scale; ISS injury severity score; BIPS bowel injury prediction score. ^†^In the post hoc tests of both variables; 1 showed statistical significance for 2 and 3.

**Table 2:** Summary of the comparison of the outcomes divided into 3 groups: ≤8 h, 8–24 h, and >24 h.

	All patients, *n* (%) (*n* = 52)	Time to operative intervention group			*p* value
		≤8 h (*n* = 33)	<8 h to ≤24 h (*n* = 13)	>24 h (*n* = 6)	
Bowel resection	31 (60)	22 (67)	4 (31)	5 (83)	0.037
LOS (days), median (IQR, 25–75)	24 (17–36)	24 (18–35)	21 (10–40)	28 (20–98)	0.321
ICU LOS (days), median (IQR, 25–75)	4 (1–12)	2 (1–12)	4 (2–26)	11 (7–14)	0.153
Mortality	3 (6)	1 (3)	2 (15)	0 (0)	0.219
Morbidity^1^	23 (44)	15 (46)	5 (39)	3 (50)	0.871
Wound complication^2^	16 (31)	9 (27)	4 (31)	3 (50)	0.540
Intra-abdominal complication^3^	9 (17)	6 (18)	2 (15)	1 (17)	0.974
Lung complication^4^	12 (23)	6 (18)	4 (31)	2 (33)	0.539
Catheter-associated complication	4 (8)	3 (9)	0 (0)	1 (17)	0.396
Acute kidney injury	2 (4)	1 (3)	1 (8)	0 (0)	0.664

SD standard deviation; LOS length of hospital stay; ICU intensive care unit. ^1^The sum of the percentages does not equal 100% because of multiple responses. ^2^Wound complication includes wound infection and evisceration. ^3^Intra-abdominal complication includes intra-abdominal abscess, leakage, enterocutaneous fistula, and intestinal obstruction. ^4^Lung complication includes pneumonia and ARDS.

**Table 3:** Comparison of outcomes between groups treated for ≤24 and those treated for >24 hours.

	Time to operation ≤24 h (*n* = 46)	Time to operation >24 h (*n* = 6)	*p* value
Male	34 (74)	4 (67)	0.655
Age (y), mean ± SD	52 ± 16	61 ± 9	0.192
Abdominal pain	43 (94)	5 (83)	0.397
Time from injury to OR (hour), median (IQR, 25–75)	4.8 (3.2–8.9)	43.8 (37.5–81)	0.001
Time from ER to OR (hour), median (IQR, 25–75)	2.7 (2–4.8)	31.1 (4.3–42.8)	<0.001
Injury mechanism			0.918
MVC	21 (46)	2 (33)	
MBC	5 (11)	1 (17)	
Pedestrian	5 (11)	1 (17)	
Falls	2 (4)	0 (0)	
Bicycle	3 (6)	0 (0)	
Others	10 (22)	2 (33)	
SBP (mmHg), median (IQR, 25–75)	110 (94–130)	103 (98–124)	0.356
Hypotension (SBP <90 mmHg)	10 (22)	0 (0)	0.582
HR (beats per minute), mean ± SD	86 ± 17	89 ± 14	0.699
GCS, median (IQR, 25–75)	15 (14–15)	15 (14–15)	0.472
CT findings (initial)			
Bowel-wall thickness	41 (89)	4 (67)	0.180
Mesenteric stranding	40 (87)	6 (100)	1.000
Free air	36 (78)	2 (33)	0.038
Free fluid	41 (89)	6 (100)	1.000
Contrast extravasation	12 (26)	0 (0)	0.316
Initial Hgb (mg/dl), mean ± SD	13.6 ± 2.3	12.1 ± 1.8	0.143
Initial WBC (10^9^/L), mean ± SD	11.8 ± 5.1	12.1 ± 7.9	0.883
CRP (*n* = 44 mg/dL), median (IQR, 25–75)	0.24 (0.08–1.07)	10.8 (0.05–24.00)	0.357
Lowest Hgb (mg/dl), mean ± SD	10.3 ± 2.1	10.3 ± 1.6	0.983
Abdominal AIS score			0.650
3	30 (65)	5 (83)	
4	16 (35)	1 (17)	
ISS, median (IQR, 25–75)	17 (10–24)		0.950
BIPS			0.823
1	4 (9)	1 (17)	
2	34 (74)	4 (66)	
3	8 (17)	1 (17)	
Bowel resection	26 (57)	5 (83)	0.382
LOS (days), median (IQR, 25–75)	24 (17–36)	28 (20–98)	0.720
ICU LOS (days), median (IQR, 25–75)	2 (1–12)	11 (7–14)	0.388
Mortality	3 (7)	0	1.000
Morbidity^1^	20 (44)	3 (50)	1.000
Wound complication^2^	13 (28)	3 (50)	0.357
Intra-abdominal complication^3^	8 (17)	1 (17)	1.000
Lung complication^4^	10 (22)	2 (33)	0.612
Catheter-associated complication	3 (7)	1 (17)	0.397
Acute kidney injury	2	0	1.000

SD standard deviation; OR operation room; ER emergency room; MVA motor vehicle collision; MBA motor bike collision; SBP systolic blood pressure in emergency department; HR heart rate in emergency department; GCS Glasgow coma scale; CT computed tomography; Hgb hemoglobin; WBC white blood cell; CRP C-reactive protein; AIS abbreviated injury scale; ISS: injury severity score; BIPS bowel injury prediction score; LOS length of hospital stay; ICU intensive care unit. ^1^The sum of the percentages does not equal 100% because of multiple responses. ^2^Wound complication includes wound infection and evisceration. ^3^Intra-abdominal complication includes intra-abdominal abscess, leakage, enterocutaneous fistula, and intestinal obstruction. ^4^Lung complication includes pneumonia and ARDS.

## Data Availability

The datasets analyzed during this study are available from the corresponding author on reasonable request.
